# The influence of city size versus urban form on land surface temperature variation and the surface urban heat island effect: A cross-city analysis of German cities

**DOI:** 10.1371/journal.pone.0340060

**Published:** 2026-02-10

**Authors:** Regan Doyle, Tobias Leichtle, Stephan Pauleit, Hannes Taubenböck

**Affiliations:** 1 CEC-Ingenieure, Gewerbegebiet An der Dornacher Straße, Feldkirchen, Germany; 2 Department Georisks & Civil Security, German Aerospace Center (DLR), Münchener Strasse 20, Weßling, Germany; 3 Strategic Landscape Planning and Management, School of Life Sciences, Technical University of Munich, Emil-Ramann-Strasse 6, Freising, Germany; 4 Department of Global Urbanization and Remote Sensing, Earth Observation Research Cluster, Institute for Geography and Geology, University of Würzburg, Würzburg, Germany; The Chinese University of Hong Kong, HONG KONG

## Abstract

This paper seeks to: 1) determine the extent of the intra-urban surface urban heat island (SUHI) effect within German cities using novel urbanization-level data with high spatial resolution, and 2) to assess the influence of city size and urban form using this data together with landscape metrics. The study uses aggregated Landsat LST data to measure the intra-urban SUHI effect across German cities of various population sizes. The first stage of the research utilizes urbanization-level data based on differing thresholds of population and building density, providing a novel method of measuring the correlation between the intra-urban SUHI phenomenon and density. The second stage assesses the impact of urban form by calculating specific landscape metrics using high-resolution land cover data, which is then clustered to identify homogeneous patterns among cities. We show that the SUHI effect can be found within all cities included in the study; however, a positive correlation between increase in city size and increase in heat stress could not be confirmed. The study shows that German urban areas are characterized by specific patterns of urban form that correspond with size, and specific urban forms particularly related to density and shape correspond with higher overall LST.

## 1. Introduction

The Surface Urban Heat Island (SUHI) effect is defined as higher temperatures within urban areas compared to their surroundings due to: increased heat absorbing surfaces from land cover changes, anthropogenic heat production from higher population densities, and development of specific air circulation patterns from the physical characteristics of the built environment [1]. The SUHI effect poses an increasing risk to human health as the resulting increased air and surface temperatures within urban environments strongly decrease levels of human thermal comfort and cause an increase in health problems and even mortality levels [2]. The SUHI effect is further exacerbated during heat waves, which have become a more common occurrence due to climate change [3]. Estimates suggest around 30% of the world population is exposed to heatwaves for at least 20 days per year [4]. In Germany, it is estimated that nearly 20,000 fatalities were associated with increased temperatures in summer between 2018 and 2020 [4].

In this paper, we aim to determine the extent of the SUHI effect in German cities and investigate the urban characteristics that influence it. In this context, Oke [5] determined an underlying relation between city size and heat island intensity, which has since been confirmed by a multiple of research studies [6,7]. Existing research has focused on heavily populated cities, as it is assumed these cities will show increased SUHI effects; however, according to UN DESA [8], in 2018, only around 20% of the world population lived in urban areas with a population greater than 5 million, while 48% lived in urban areas of fewer than 500,000 people (classified as urban settlements). Based on these assertions, there is need for further research into the SUHI effect and its extent within small-medium sized cities. In Germany, 78% of the population resides within urban areas but only about 17% of the total population lives within cities having a population greater than 500,000 [9], indicating that a large percentage of the urban population resides within urban settlements. This research seeks to fill this gap in existing literature by examining German cities of various population ranges for comparison and measurement of the intra-urban SUHI effect.

The spatial variation of temperature in urban areas is a result of complex interactions, such as land use, surface and air temperature gradients, and surface energy exchange [10]. As such, earth observation data is crucial for the study of the SUHI, as remote sensing technology allows for a detailed investigation of the intensity and spatial distribution of the land surface temperature (LST) [11]. LST measurements provided by remote sensing analysis have proven indispensable for the assessment of the SUHI as well as land use/land cover (LULC) change and their relationship within existing research [12]. It is important to note that surface temperature is not equivalent to air temperature (T_a_); however, studies have shown a strong correlation between T_a_ and LST [13], though additional factors such as geographical location and land cover significantly impact the relationship [2]. While research has argued that T_a_ is more directly relevant for public health, the limited data and sensors for monitoring T_a_, particularly in high-density built areas, limits research capabilities and are insufficient in providing spatial details in an area-wide manner [14]. Compared to temperature values collected from weather stations, thermal imagery provides full spatial coverage at various spatial and temporal scales, and is better suited to illustrate hot and cold areas, particularly in urban environments [15]. The primary limitations for remote sensing data application are limited spatial coverage and inaccuracies due to clouds or other factors [16]. Despite potential errors in data, remote sensing has been found to be largely accurate (between 85 and 95%) overall and continues to improve with advancements in technology [17].

The primary cause of the SUHI effect relates to the structural and LULC differences between urban and rural areas, as the shift from natural land to impervious surface associated with urbanization alters the thermal transference of the environment, resulting in higher overall temperatures [1]. As cities expand, the increase in LULC change further intensifies the differences between urban and rural structural characteristics, exacerbating the SUHI phenomenon [18]. Despite the heterogeneity of land surface characteristics across urban environments as it shifts from more rural to more urban, the conventional approach to measure the SUHI effect has been to classify a site as ‘urban’ or ‘rural’ based on perfunctory administrative boundaries and measure temperatures at representative locations. Such oversimplification of ‘urban’ and ‘rural’ is often ambiguous and cuts out regions and populations along the continuous gradient between the two classifications, reducing the accuracy of data findings [19]. To correspond with changes in land surface characteristics, research must seek to define regions based on uniform characteristics (e.g., surface cover, surface structure, human activity, etc.) to provide objective interpretation of LST variation [1]. A recent study by Taubenböck et al. [19] sought to fill this gap by using a multimodal method to systematically map the degree of urbanization in terms of population as well as building density and type. By utilizing this novel urbanization-level data to measure the intra-urban SUHI effect, a more detailed analysis that overcomes the oversimplification of ‘urban’ and ‘rural’ can be achieved within this research.

Existing literature has consistently found that the physical characteristics of a city, such as size, shape and spatial layout significantly impact variation in LST [6,7,20–23]. A large quantity of research has found positive correlations between built-up, or impervious land, and areas of increased temperature within urban environments, while vegetation and water bodies were found to have a cooling effect [1,18,24,25]. Additionally, aspects such as density and centrality as well as city shape and regularity of built-up surfaces have been found to increase or decrease the SUHI effect. For example, several studies have noted positive correlations between circular, or more regularly shaped urban areas and increased SUHI effect, whereas urban areas with a more complex or irregular shapes negatively correlate to average temperatures [22,23,26]. A study by Yue et al. [23] found that an increase in the shape complexity of an urban area may enhance ventilation between surrounding environments. Similar results were determined by Su et al. [26], who found that more rotund shaped cities with high levels of density lead to increased intensity in the SUHI phenomenon. Based on these findings, it could be asserted that urban land cover and form, rather than city size has a greater impact on the SUHI effect, and therefore, the impact of urban form across cities of various scales provides potential insight as to the validity of said assertion and how patterns in urban form impact LST variation within German urban environments.

Based on the findings of existing literature, variations in temperature can be found within the boundaries of a single urban environment, indicating a contextual analysis of the SUHI phenomenon is required to understand what potential location-specific factors have resulted in increased temperatures within an urban environment. As such, this study seeks to understand the SUHI effect within German cities to potentially identify any spatial patterns or similarities. This quantitative information supports planners and policymakers in developing possible mitigation measures against future temperature rises in order to efficiently reduce potential health risks for urban residents. The objective of this research is to determine; 1) the extent of the SUHI effect and how city size may influence the effect within Germany; and 2) how urban form influences SUHI patterns in German cities. To do so, German cities of various sizes were examined to determine correlations among urban characteristics that result in higher LST.

It is hypothesized that the SUHI effect will be found within all cities in Germany, despite their size; however, based on existing literature, it is assumed the effect will increase with city size. The use of the urbanization-level data provides a novel method of examining how much the SUHI effect increases based on density within a city. Therefore, it is assumed the areas of highest density within the largest cities in Germany will experience the highest LST values as a result of the SUHI effect. The examination of urban form through landscape metrics combined with the analysis of SUHI based on urbanization levels is a novel approach; however, it is assumed that urban form will help identify potential patterns in the SUHI effect within German cities.

## 2. Materials and methods

The research utilizes statistical and GIS analysis based on remote sensing data and consists of an intra-urban SUHI analysis using urbanization level-data, followed by a comparative analysis using land cover data.

We assume that an intra-urban SUHI effect can be found in all cities; however, the analysis will determine its differences between cities based on urbanization levels. To do so, the statistical relationships between average mean and maximum LST and urbanization levels are analyzed to identify any potential patterns of the SUHI effect. Furthermore, we investigate whether city size may influence SUHI measures using population groupings. The use of land cover data allows for comparison of the urban form of each city to potentially explain spatial patterns of the SUHI effect. To do so, the study uses specific indicators of urban form based on landscape metrics. The cities are then clustered based on these indicators and the results are compared to mean and maximum LST measures. This research is novel in two key aspects: first, such a comparative study including medium- and small-sized cities has not been previously carried out in Germany. Second, the novel urbanization data allows the research to overcome articifcal boundaries of administrative units for a structurally consistent examination of the intra-urban SUHI phenomenon.

### 2.1. Study area

**Fig 1:**
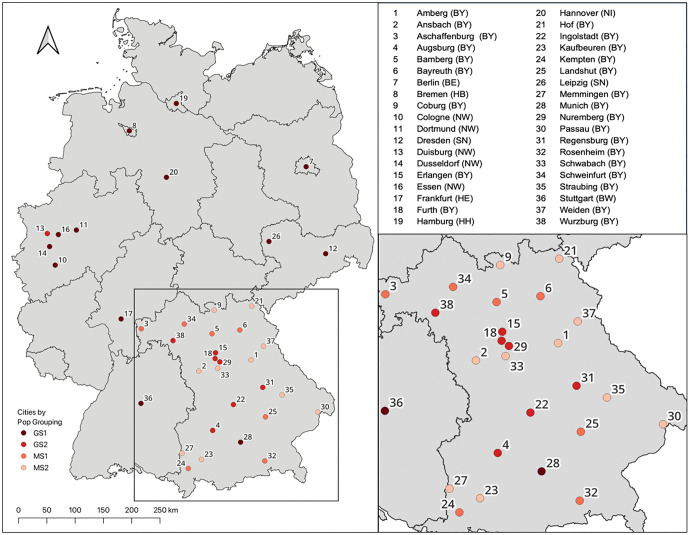
Selected German Cities – the 15 largest cities of Germany as well as all urban districts of Bavaria. Citation: Map data is available at USGS National Map Viewer (public domain): http://viewer.nationalmap.gov/viewer/.

To carry out the analysis, the 15 largest cities in Germany have been chosen for analysis. In addition, all Bavarian urban districts, or *Kreisfreie Staedte*, are included in the study to add smaller towns and cities to the sample. As such, an analysis of the largest urban areas of Germany combined with medium and smaller sized cities – according to the BBR [27] urban classifications – can provide important insight into the variables that impact LST variation within cities of various population classes. Thisis crucial for urban planners given the large percentage of the German population living in medium-small sized cities. Therefore, a total of 38 cities are included in the analysis, as illustrated in Fig 1 below.

Bavaria was chosen for the analysis, not only because it is the largest state in Germany, but also because the rural nature of the state provides a large number of medium-small sized cities for analysis and meets the research requirements regarding variation in urbanization as well as land cover typologies. The selection allows the study to examine not only the largest cities in Germany, but also medium-small cities of the largest federal state; thereby, achieving a high variability of city types.

For the comparative analysis, the cities are divided into population groupings based on German ‘City and Municipality Types’ as defined by the German Federal Institute for Construction and Regional Planning (BBR) [27]. The population groupings used within the analysis, as well as their corresponding populations are listed in Table 1.

**Table 1:** City population groupings.

Population Grouping (DE)		Population Grouping (ENG)	Population
Grossstadt 1	(GS1)	Large city 1 (GS1)	> 500,000
Grossstadt 2	(GS2)	Large city 2 (GS2)	100,000–499,999
Mittelstadt 1	(MS1)	Medium-sized city 1 (MS1)	50,000–99,999
Mittelstadt 2	(MS2)	Medium-sized city 2 (MS2)	< 50,000

### 2.2. Data collection

**Fig 2:**
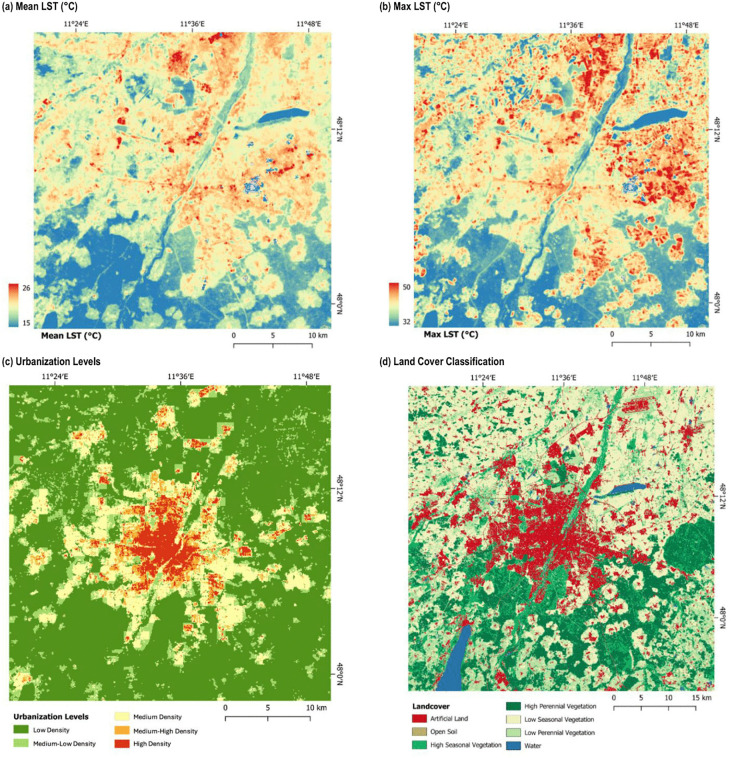
Examples of data used in the research (Munich). Citation: LST data is available at Landsat: http://landsat.visibleearth.nasa.gov/. All data was prepared by DLR.

The various datasets were spatially harmonized using a 1-hectare (ha) INSPIRE grid database [28]. This grid provides the basis for uniform transformation of all input data to reduce noise and improve model robustness in subsequent analyses. The following data is included:

- Land Surface Temperature (LST): LST was obtained from Landsat 8 and 9 Operational Land Imager (OLI) and Thermal Infrared Sensor (TIRS) images with a spatial resolution of 30 meters from the years 2013–2023. Landsat data captures the Earth’s surface in a 16-day repeat cycle with a swath coverage of 185 x 185 km [29]. The last ten years were included to compensate data inconsistencies due to differing acquisition dates of neighboring orbits as well as additional data gaps due to cloud cover, thereby providing an accurate average for analysis. A total of 10,822 Landsat satellite images were collected and processed. Statistical aggregates were computed to determine the average mean and maximum LST for all 38 cities in the analysis covering the years 2013–2023 (see Figs 2a and 2b for an example).
- Urbanization Levels: The urbanization levels used in the analysis are based on a study by Taubenböck et al. [19]. As the study assumes the ‘*urban is a form of higher density*’ [19, p. 3], areas of increased urbanization consist of increased density of population and built space; therefore, the levels were calculated based on the parameters of building density, population density, and building typology determined at a spatially continuous and consistent grid of 1 hectare. A probability-based approach was then applied to combine the different parameters and five urbanization level thresholds are produced, which include: High Density (HD), Medium-High Density (MHD), Medium Density (MD), Medium-Low Density (MLD), and Low Density (LD) (see Fig 2c).
- Land Cover: Land cover data is taken from the study of Weigand et al. [30]. The data features high spatial resolution of 10 meters as well as area-wide coverage of the entire country of Germany. The data was created using images collected from Sentinel-2 satellite data and includes seven thematic land cover classes: artificial land or built-up land (AL), open soil (OS), high seasonal vegetation (HSV) and high perennial vegetation (HPV), low seasonal vegetation (LSV) and low perennial vegetation (LPV), and water (W) (for more details see Table 2 and Fig 2d). For analysis, percentages of each land cover found within each grid cell are calculated and combined into the grid database.

**Table 2:** Land cover classes.

Classification	Description
Artificial Land (AL)	Built-up or impervious land (e.g., buildings, roads, etc.)
Open Soil (OS)	Areas of land devoid of vegetation (e.g., dirt patches, or cropland with no current growth)
High Seasonal Vegetation (HSV)	Deciduous tree cover (e.g., broadleaf forests or fruit tree crops)
High Perennial Vegetation (HPV)	Evergreen tree cover (no change throughout the year)
Low Seasonal Vegetation (LSV)	Croplands with seasonal variation
Low Perennial Vegetation (LPV)	Pastures or grasslands (no change throughout the year)
Water (W)	Bodies of water (e.g., rivers, lakes, seas, etc.)

### 2.3. Analysis of SUHI effect

To determine the extent of the SUHI effect and investigate patterns that influence the SUHI effect within German cities, a statistical comparative analysis of LST data based on urbanization levels has been carried out. For the comparative analysis of cities, the established administrative boundaries of the selected cities are utilized as the most practical way to define and assign raster cells and subsequent data to each city; however,the LST data is summarized in terms of mean and standard deviation (SD) by the particular urbanization level. To further assess the difference in LST value between cities of various population groupings, the average effect of the urbanization level, i.e., the difference between HD and other urbanization type (x), on the surface temperature is evaluated as


$$\Delta LST(x)={LST}_{HD}-{LST}_{x}$$

where ${LST}_{x}\;$ is the average Landsat LST of all grid cells of urbanization level x per city. Positive values indicate a higher temperature in the HD area with differences increasing as areas become less dense. For x = LD, ΔLST(x) coincides with the surface urban heat island intensity (SUHII). The effects on ΔLST(x) for all urbanization levels are examined first for all 38 cities and then the same procedure is applied in evaluating the effect for each population grouping to determine how city size influence SUHI patterns in Germany.

### 2.4. Characterization of cities by urban form

To determine how urban form influences SUHI patterns in German cities, we analyze the 38 cities based on landscape metrics established by Schwarz [31], which largely represent the most relevant indicators of urban form. A k-means cluster analysis is used to determine if commonalities can be identified based on the urban form indicators. These clusters are then compared with LST values to determine which aspects of urban form result in higher temperatures, and vice versa. These results enable the confirmation or rejection of the hypothesis that urban form influences the SUHI effect. By comparing cities based on population groupings, it is also possible to determine which aspects of the urban form common to specific cities – potentially related to city size – impact LST variation. We specifically focus on maximum LST given the potential risk of heat stress events on human health.

#### 2.4.1. Landscape metrics.

Landscape metrics are defined as “algorithms to quantify the spatial structure of patterns within a defined geographic area” [32]. The selected landscape metrics were identified from existing studies as these indicators were found to be applicable to a large number of cities for comparison while avoiding high correlations that may result in redundant variables [31,33]. The selected metrics include a sequence of quantitative indices that represent differing physical characteristics of the urban setting: total impervious area (TSA), edge density (ED), mean patch size (MPS), number of patches (NP), compactness index of the largest patch (CILP), area weighted mean shape index (AWMSI), and centrality index (CI) (seeTable 3).

**Table 3:** Urban form indicators, measures and definitions.

Indicator	Measurement (per city)	Definition	Source
Total Sealed Area [TSA]	$TSA\;=\;\sum_{i=\text{1}}^{n}a_{i}$ i = 1, …, n sealed patches, $a_{i}$ = area (m^2^) of patch i.	The absolute spatial extent of urban/artificial (impervious) area (m^2^).	[34]
Edge Density [ED]	$ED\;=\;\frac{\sum_{i=\text{1}}^{n}e_{i}}{\sum_{i=\text{1}}^{n}\;a_{\text{j}}}$ i = 1, …, n sealed patches, $e_{i}$ = total length (m) of edge/perimeter of patch i, $a_{i}$ = area (m^2^) of patch i.	Length of the edge relative to the area of the patch (1/m)– the higher the value, the more ragged the patch.	[35]
Mean Patch^a^ Size [MPS]	$MPS\;=\;\frac{\text{1}}{n}\sum_{i=\text{1}}^{n}a_{i}$ i = 1, …, n sealed patches, $a_{i}$ = area (m^2^) of patch i.	Average size of sealed patches (m^2^).	[31]
Number of Patches [NP]	NP=n i = 1, …, n sealed patches.	Number of sealed patches.	[31]
Compactness Index of the Largest Patch [CILP]	$CILP\;=\;\frac{\text{2}\pi \sqrt{a_{\left(n\right)}/\pi }}{e_{\left(n\right)}}\;$ $a_{\left(n\right)}$= area of largest patch, $e_{\left(n\right)}$= edge/perimeter of largest patch.	Indicates compactness of largest sealed patch (between 0 and 1) – higher value indicates more compact with a more regular shape (full circle has index value of 1).	[33]
Area Weighted Mean Shape Index [AWMSI]	$AWMSI=\;\sum_{i=\text{1}}^{n}[\frac{\text{0}.\text{25}e_{i}}{\sqrt{a_{i}}}\;\;\times \;\;\frac{\;a_{i}}{\sum_{k=\text{1}}^{n}\;a_{\text{k}}}]$ i = 1, …, n sealed patches, $a_{\text{i}}$ = area (m^2^) of patch i, $e_{i}$ = total length (m) of edge/perimeter of patch i.	Represents the shape irregularity of the patches (scores >0) – the higher the value, the more irregular the sealed patches, index for each patch is weighted by its relative area.	[33]
Centrality Index [CI]	$CI\;=\;\frac{\frac{\text{1}}{n-\text{1}}\sum_{j=\text{1}}^{n-\text{1}}D_{j}}{\sum_{i=\text{1}}^{n}a_{i}\;/\;\pi }$ i = 1, …, n sealed patches, j = 1, …, n-1 sealed patches without largest patch, $D_{j}$ = distance of centroid of patch j to centroid of the largest patch.	The average distance of sealed patches from the largest sealed patch relative to the radius of a circle with area equal to the total sealed area (scores >0).	[33]

Some indicators are measured differently based on raster or vector data [36]. The original rasters were used for simplified calculation purposes.

^a^ A patch is defined as neighboring cells belonging to the same class.

These indicators represent complementary dimensions of urban form, i.e., city size, compactness, centrality, complexity, porosity and density. TSA represents the overall size of the city by providing the average size of the sealed (impervious) area, i.e., AL. ED assists in measuring complexity as well as porosity as it measures the irregularity or smoothness of patch edges, which indicates the relationship between the impervious area and surrounding classes. MPS and NP combined with CI represent density, as MPS and NP indicate the number and size of impervious patches, while CI determines their relative proximity. CILP measures compactness by measuring the overall shape of the largest patch, which accounts for the bulk of the urban area for most cities. AWMSI represents complexity by measuring the irregularity of the patch shape, with higher values indicating more irregular shapes. Finally, CI represents centrality and density, as it measures the average distance of dispersed urban areas to the city center, and therefore, the overall shape of the city.

The landscape metrics were computed based on the artificial land (AL) (i.e., impervious surfaces) from the land cover data, as these areas represent urban or built form of the city. All indicators were normalized using the z-transformation for mean 0 and standard deviation (SD) 1 to ensure comparability.

#### 2.4.2. K-means clustering.

To compare the 38 German cities according to their urban form, a cluster analysis is computed using the landscape metrics as indicators of urban form. The k-means cluster algorithm was chosen for the analysis as it is the most widely used technique due to its simplicity and ability to group large quantities of data with low computing time [37]. The k-means algorithm requires that the number of clusters must be defined prior to computing. To determine the optimum number of clusters, the Elbow method is employed, which is a visual method comparing the within-cluster sum of squares (WCSS) for each cluster count to determine the optimal number of clusters [37].

## 3. Results

### 3.1. Analysis of SUHI effect

**Fig 3:**
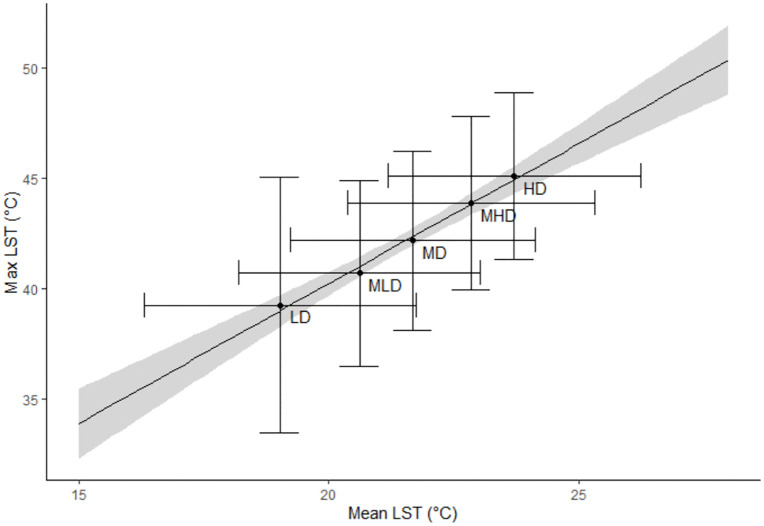
Average maximum LST of five urbanization levels compared with their average mean LST for all 38 cities. Bars denote standard deviation (SD).

The average maximum LST of the five urbanization levels in relation to their average mean LST across all 38 cities is illustrated in Fig 3. The analysis confirms the hypothesis that areas of HD have a higher overall mean and maximum LST than the remaining lower density urbanization levels. A linear increase of LST with urbanization levels could be observed. Between the HD and LD levels is an absolute difference of about 6°C for both the mean and maximum LST.

**Fig 4:**
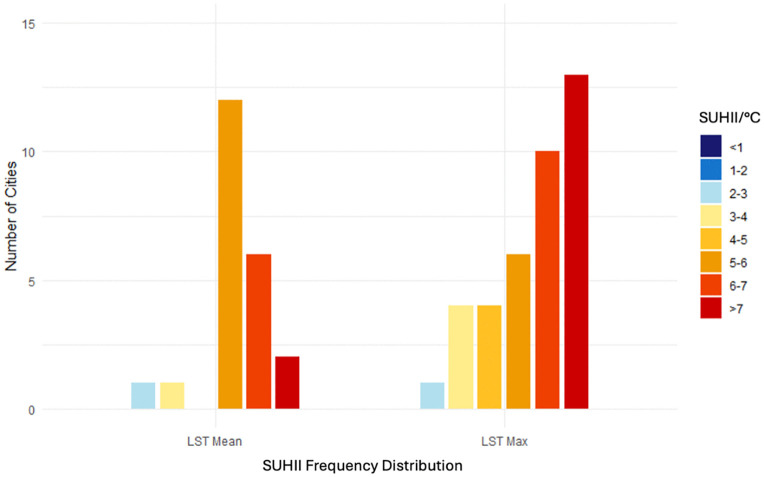
SUHII (i.e., difference between High Density (HD) and Low Density (LD) urbanization levels) of mean and maximum LST for 38 cities in Germany.

The SUHII frequency distribution of mean and maximum LST among the 38 cities is illustrated in Fig 4. The analysis shows that for the mean LST values, the majority of cities have a difference of 5-6°C between HD and LD. For the maximum LST values, most cities have a difference of greater than 7°C between HD and LD, though the cities are more evenly distributed among the SUHII categories. The significant difference between LD and HD, particularly for maximum LST, indicates urban areas of higher density heat up significantly more than other urbanization levels during heatwaves.

The analysis comparing average maximum LST of the five urbanization levels to their average mean LST by city population groupings found similar results to the overall analysis of all 38 cities. LST mean and maximum values are consistently higher in HD than in LD regardless of population size (Table 4). Interestingly, comparison of LST for HD and LD in cities of different population size reveal that mean and maximum LST are higher in smaller cities than large cities (Table 4). Therefore, an initial assumption of positive correlation between LST and population size cannot be confirmed.

**Table 4:** Averages of absolute LST by population groupings for mean (a) and maximum (b) LST.

(a) Mean LST					(b) Max LST				
Pop Class	Avg. HD LST	Avg. LD LST	Avg. ΔLST(LD)	Total Mean LST	Pop Class	Avg. HD LST	Avg. LD LST	Avg. ΔLST(LD)	Total Max LST
**GS 1**	23.44	18.77	4.67	20.4	**GS 1**	44.71	38.73	5.98	40.6
**GS 2**	24.94	19.67	5.27	21.2	**GS 2**	47.11	39.76	7.35	41.7
**MS 1**	25.21	19.15	6.06	20.5	**MS 1**	47.39	40.05	7.34	41.7
**MS 2**	25.12	19.40	5.72	20.4	**MS 2**	46.63	40.73	5.90	41.7

**Fig 5:**
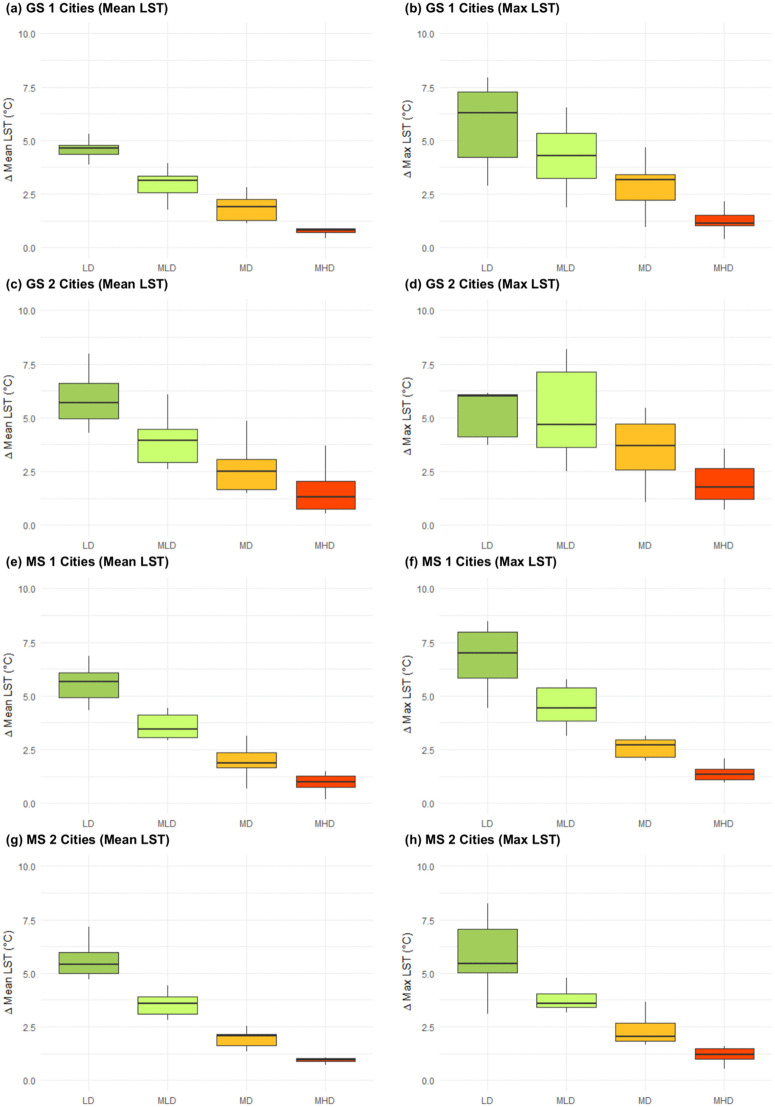
Average differences (median upper and lower quartile) in LST (Δ𝐋𝐒𝐓) between High Density (HD) and other urbanization levels (LD, MLD, MD, MHD) respectively. **(a)** GS 1 mean LST; **(b)** GS 1 max LST; **(c)** GS 2 mean LST; **(d)** GS 2 max LST; **(e)** MS 1 mean LST; **(f)** MS 1 max LST; **(g)** MS 2 mean LST; **(h)** MS 2 max LST.

For each group, the difference between HD and LD levels is illustrated in Fig 5. The analysis indicates greater variation in the data for maximum LST compared to mean LST; however, the variation is not as significant as density increases, reducing potential inaccuracies in the results in HD areas. Unexpectedly, the median ΔLST for the mean LST in GS 1 cities (Fig 5a) is lower than the other city population groupings, indicating that either the average mean temperature is warmer in less dense areas or that the average temperature in more dense areas is cooler. These LST values do not correspond with the assumption that greater density results in higher average LST, as Table 5 illustrates the proportionate cover of each urbanization level for the different population groupings to compare LST differences. As the table shows, GS1 cities have the highest percentage HD cover and lowest percentage LD cover. A similar table providing the percent cover of urbanization level by city is found in S1 Table, which illustrates that the percent cover of HD increases with city population while LD decreases.

**Table 5:** Population groupings and proportionate % cover by urbanization level.

Pop Class	% HD	% MHD	% MD	% MLD	% LD
GS 1	9.13	8.36	23.73	18.41	40.37
GS 2	5.96	7.34	20.65	16.88	49.17
MS 1	3.76	5.35	18.08	15.20	57.61
MS 2	1.71	3.01	13.25	16.53	65.51

### 3.2. Characterization of cities by urban form

#### 3.2.1. Landscape metrics.

The correlation values between the landscape metrics are provided inTable 6. The correlations with maximum LST values are included to provide insight on the influence of landscape metrics on LST values. The analysis specifically focuses on maximum LST, in order to predict areas with the highest exposure due to their physical form.

**Table 6:** Correlation values of urban form indicators.

	TSA	ED	MPS	NP	CILP	AWMSI	CI	MEAN LST	MAX LST
**TSA**	**1**	0.029	0.069	0.920***	−0.779***	0.966***	−0.469**	−0.017	−0.214
**ED**		**1**	−0.838***	0.239	0.016	0.037	0.482**	−0.336*	−0.436**
**MPS**			**1**	−0.228	−0.153	0.074	−0.552***	0.261	0.256
**NP**				**1**	−0.670***	0.864***	−0.300.	−0.058	−0.250
**CILP**					**1**	−0.860***	0.633***	0.046	0.292.
**AWMSI**						**1**	−0.552***	−0.034	−0.255
**CI**							**1**	−0.116	−0.082
**MEAN LST**								**1**	0.662***
**MAX LST**									**1**

Significance codes: 0 ‘***’ 0.001 ‘**’ 0.01 ‘*’ 0.05 ‘.’ 0.1 ‘’ 1

Total Sealed Area (TSA); Edge Density (ED); Mean Patch Size (MPS); Number of Patches (NP); Compactness Index of the Largest Patch (CILP); Area Weighted Mean Shape Index (AWMSI); Centrality Index (CI)

As indicated in Table 6, very few of the urban form indicators significantly impact LST variation. The results show a significant negative correlation between ED and both mean LST (-0.336*) and maximum LST (-0.436**), indicating that the increased complexity of the shape of artificial land patches with greater edges provide cooler temperatures. Additionally, a relatively high positive correlation can be seen between CILP and maximum LST (0.292.), indicating that the increased compactness of the urban center (or the largest patch) having a more regular (rounded) shape correlate to higher maximum LST. It is important to note that each city was found to have only one ‘largest patch’ – equating to an urban center. This corresponds with the ED values and may indicate cities with irregular and less compact urban centers have reduced effects during heat stress events.

#### 3.2.2. Clustering and characterization of cities.

**Fig 6:**
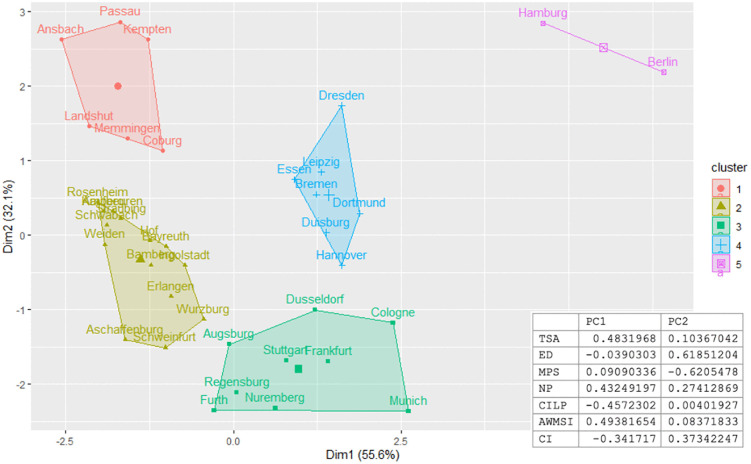
Final output of five calculated clusters along the first two principal components. The table on the bottom right shows the contributing weigths of landscape metrics.

The elbow method calculations determine the optimal number of clusters to be 5 for the indicators used. Table 7 shows the results for the variance analysis and the comparison of means to test the differences between the clusters. Fig 6 illustrates how the principle components are distributed among the clusters.

**Table 7:** Variance of urban form indicators by cluster.

Urban form Indicators					
	Cluster 1	Cluster 2	Cluster 3	Cluster 4	Cluster 5
*N*	6	14	9	7	2
	**Mean (SD)**	**Mean (SD)**	**Mean (SD)**	**Mean (SD)**	**Mean (SD)**
**TSA**	−0.66 (0.02)	−0.61 (0.1)	0.3 (0.64)	0.43 (0.28)	3.33 (0.98)
**ED**	1.62 (0.82)	−0.33 (0.46)	−0.99 (0.43)	0.24 (0.35)	1.02 (0.38)
**MPS**	−1.17 (0.35)	0 (0.47)	1.42 (0.46)	−0.58 (0.49)	−0.84 (0.48)
**NP**	−0.44 (0.06)	−0.52 (0.06)	−0.16 (0.28)	0.55 (0.44)	3.73 (0.16)
**CILP**	0.55 (0.51)	0.92 (0.49)	−0.6 (0.54)	−1.08 (0.11)	−1.6 (0.1)
**AWMSI**	−0.65 (0.2)	−0.69 (0.15)	0.32 (0.66)	0.7 (0.22)	2.94 (1.35)
**CI**	1.37 (1.06)	0.35 (0.57)	−0.86 (0.37)	−0.58 (0.73)	−0.65 (0.27)

Total Sealed Area (TSA); Edge Density (ED); Mean Patch Size (MPS); Number of Patches (NP); Compactness Index of the Largest Patch (CILP); Area Weighted Mean Shape Index (AWMSI); Centrality Index (CI)

**Fig 7:**
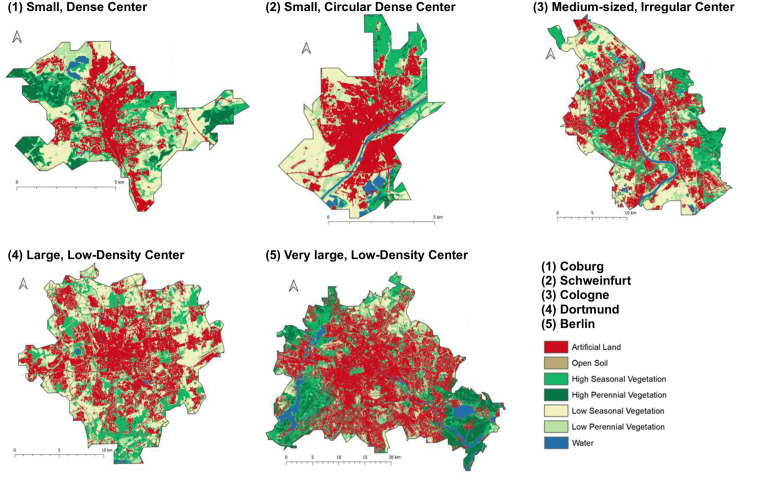
Examples of cluster types 1–5, visualized using land cover data. Citation: All data was prepared by DLR.

The five clusters obtained in this study can be characterized as follows, visualized examples based on land cover can be found in Fig 7:

Cluster 1: *Small cities, dense center*: Cities classified into cluster 1 are characterized as small cities with low total impervious cover (AL). They have a very compact urban center (largest patch of AL surface) with few patches of impervious surface disconnected from the center. The relative shape of the largest patch and surrounding patches may vary and be more circular or irregular, but they tend to have ragged edges.

Cluster 2: *Small cities, circular dense center* has by far the largest number of cities of any cluster at 14, indicating many smaller German cities have developed in a similar manner. Cities classified into cluster 2 are small cities, with similar total impervious cover as cluster 1; however, they have even fewer surrounding patches and larger average patch size, indicating a larger urban center and greater connectivity.

Cluster 3: *Medium-sized cities, irregular center* is made up of medium and larger sized cities. The low centrality and irregular shape of surrounding patches indicates high connectivity with high compactness.

Cluster 4: *Large cities, low-density center* have a rather large impervious area with many patches, indicating larger cities that are more spread out and disconnected. The patches are irregularly shaped and relatively close together, but smaller and more dispersed than cluster 3.

Cluster 5: *Very large cities, low-density center* only consists of two cities – Berlin and Hamburg. These cities are the largest of the 38, which corresponds to the highest amount of impervious surface. The cities are characterized by a high number of patches that are smaller in size but very close together and irregularly shaped with very ragged edges. This indicates a city that is largely built up with impervious surface that is spread out across the city at frequent intervals.

#### 3.2.3. Impact of urban form on LST variation.

To analyze the impact of urban form on LST variation, the average total mean and maximum LST of each city was calculated (see S2 Table). These values are then averaged once again based on the clusters as illustrated in Table 8 below.

**Table 8:** Comparison of clusters to average mean and maximum LST.

Cluster		Mean LST M (SD)	Max LST M (SD)
1	Small, dense center	19.89 (2.07)	40.33 (1.548)
2	Small, circular dense center	20.93 (1.665)	**42.28 (1.96)**
3	Medium-sized, irregular center	**20.96 (1.376)**	41.80 (1.415)
4	Large, low-density center	20.11 (1.106)	40.32 (1.542)
5	Very large, low-density center	20.54 (1.324)	40.14 (1.907)

As indicated in Table 8, both average mean and maximum LST values vary based on the assigned cluster; however, the values are relatively similar across the clusters and the differences between clusters are less than the SDs. Cluster 3 has the highest average mean LST, followed closely by cluster 2, though all the clusters apart from cluster 1 have an average mean LST between 20 and 21°C. Unexpectedly, the maximum LST does not correspond to the mean LST, as cluster 2 has the highest maximum LST of 42.28°C, followed by cluster 3 with 41.80°C. This indicates that the urban form affects the mean and maximum LST differently. The other 3 clusters have an average maximum LST between 40 and 41°C, with cluster 5 (containing the two largest German cities) having the lowest average maximum LST at 40.14°C.

## 4. Discussion

### 4.1. Extent of the SUHI effect and the influence of city size in Germany

As was originally hypothesized, the intra-urban SUHI phenomenon can be observed in every city within the study. The cities have a significant difference in temperature between HD and LD defined areas with LST values increasing as density increases. Therefore, the hypothesis regarding the existance of the SUHI effect as well as positive correlation between higher LST values and increase in density within the selected cities can be confirmed.

For most cities in the study, the temperature difference between the densest and least dense spaces is around 5-6°C for the yearly average (mean LST) and more than 7°C for the highest average (maximum LST) temperature. This corresponds with existing literature [1,18,21] in that increased density and impervious land coverage associated with urbanization increase the SUHI effect. While this is not a novel finding, the approach offers a potential new methodology to measure the SUHI effect based on levels of density within a city rather than traditional administrative boundaries or buffering. As administrative or buffer units are structurally very hetereogeneous, the urbanization level data provides a local and structurally more consistent understanding of the SUHI phenomenon. It should be noted the aggregation of the urbanization level data makes it unsuitable for more detailed analysis of the urban form. For example, when compared with the land cover data, the urbanization data does not explain how underlying factors such as land cover typologies specifically impact LST variation independently; therefore, more detailed landcover data is recommended for comprehensive analysis at the city level.

By comparing the levels of urbanization, the research was able to compare the intra-urban SUHI phenomenon within cities of various sizes and found unexpected results. We initially assumed that larger cities (GS 1) would exhibit the largest SUHI effect, having higher average mean and maximum LST values than cities of smaller population groupings (GS 2, MS 1, MS 2) due to larger accumulations of impervious surfaces; however, this hypothesis must be declined in the context of the study. Larger German cities have a lower average mean and maximum LST across all urbanization levels than cities in the smaller population groupings. This is in stark contrast to findings of existing literature, which commonly found a positive correlation between the expansion and growth of cities and increased SUHI effects [5–7,21,22,26].

One possible explanation is that German cities are relatively small compared with cities in other parts of the world, such as Asia and the Americas – a common focus of many existing studies. The largest German cities would be considered medium-small according to the UN definition [8]. Further research including additional German cities and other European countries may provide additional insights. Another possible explanation is that German cities have developed in a significantly different manner from the sampled cities in other studies due to their long history. Based on the findings, it is likely, that urban form, such as building density and regularity, has a greater influence on the SUHI effect in German cities than initially hypothesized, which corresponds with findings by Li et al. [20], who found that in medium-small sized cities in China (equivalent in population to GS1 cities in Germany), landscape configuration potentially has a greater impact than impervious surface coverage. Further investigation into the night-time SUHI effect of German cities may prove otherwise, as a study by Liu et al. [22] found that the size (compared to landscape configuration) of an urban area has a greater impact on the SUHI effect at night. This study also found that the internal structures of cities in combination with urban size likely influence the SUHI effect, and therefore, SUHI is relative.

There is research that proposes densification of urban spaces to reduce GHG emissions associated with travel and to protect existing natural landscapes [38]. On the other hand, such urban planning can significantly increase the SUHI effect, increasing exposure to heat stress. Further in-depth analysis of the SUHI effect on a city level may provide more detailed information, such as specific areas for urban planners to focus their efforts. In-depth analysis would also assist in identifying more specific potential heat sources and sinks within German cities to aid urban planners in developing mitigation strategies. While a large-scale analysis such as in this study does provide evidence that the SUHI effect is visible within all German cities, it may be the case that vulnerability to heat is not equal for all city residents with risk being higher by different degrees of exposure, as was found to be the case in a study of US cities by Hsu et al. [39]. Understanding where the effect is the highest and who is impacted is essential to support inclusive adaptation measures.

### 4.2. Influence of urban form

The study has found that variation in LST can be explained by urban form, which is in line with findings from previous studies [22,23,26,40]. When comparing the derived clusters to the average total mean and maximum LST of the cities, the difference in LST values between the clusters is not significant. This was particularly the case for mean LST, likely due to variation in the data and the use of city averages. Greater variation is found between the clusters when comparing maximum LST values, which provides valuable insight into the SUHI effect during heat wave events that exponentially increase risk for more vulnerable urban residents [39]. While the clusters do not identify significant variation in LST, they have confirmed that certain characteristics of urban form can be positively identified that are associated with higher average LST values, specifically the compact and circular nature of urban centers with high connectivity. These findings are similar to those in a study of European cities using geographically weighted regression analyses by Mashhodi and Unceta [40], which found that almost 70% of LST inequality could be explained by urban form, particularly impervious surfaces, density, compactness, and vegetation. The clustering method has proven useful in characterization of the urban form, which was not possible in the analysis of the urbanization levels. The combination of both methods has provided insight into the extent of the intra-urban SUHI effect, and how urban form may influence the SUHI effect during heat wave events.

Urban form differs significantly even within national borders likely due to planning regimes and historical development, as well as terrain and natural features such as rivers or mountain ranges; however, we can see some commonalities based on city size, which is consistent with previous studies that found a significant influence of urban size on urban form development [41]. Similar to findings by Liu et al. [22], this study has found that very large German cities tend to have a more complex urban shape with less evenly dispersed built areas and larger clusters of impervious surface, identified as cluster 5 in the k-means cluster analysis. This urban form is likely associated with increasing irregularity of impervious patch shape as the city expands. While it was initially hypothesized that the higher overall amount of impervious surface would result in higher average LST values, the findings indicate that patch dispersal and irregular shape associated with the historical development has exerted a cooling effect on the urban environment, as cluster 5 cities were found to have the lowest average maximum LST values based on the city total average. These findings are consistent with a study of Chinese cities by Yue et al. [23], which found that change of urban contiguity and increase in shape complexity provide an extra cooling effect, reducing the SUHI effect. Additionally, while there are many patches of urban surface in proximity, there is a high level of fragmentation, with scattered patches of vegetation between the urban surfaces. As green spaces are known to cause a cooling effect and alleviate the effects of heat islands [1,18,24,25], the scattered vegetation between urban spaces is beneficial for reducing thermal homogeneity associated with heat waves and the SUHI phenomenon.

The analysis confirms that the urban form of clusters 2 and 3 result in higher average LST than the remaining clusters. As we know from the characterization of the various clusters, cities classified as cluster 2 have a large circular-shaped urban center that is very condensed, with few surrounding areas classified as impervious. Previous studies [22,26] found similar results, in that LST is positively correlated with urban form factors such as compactness, complexity and shape. As suggested by these existing studies [22,26], this urban form likely prevents ventilation, as warm air is trapped within urban areas and cool air from surrounding environments is unable to circulate. Upon closer examination of the structure of each city, we find highly compact urban centers, with little vegetation between these spaces. Additionally, a large portion of the vegetation immediately surrounding the impervious surface is classified as low seasonal or low perennial vegetation. Areas of decreased vegetation cover, such as open or agricultural land have been found to correspond with higher temperatures, particularly during the daytime [25]. These surrounding vegetative types likely do not offer relief during heat stress events and may help explain higher maximum LST values. Cities classified as cluster 3, while larger on average than cities in cluster 2, did share similar characteristics, namely the compact nature of impervious surfaces. There are more cases of vegetation scattered between patches; however, this is largely due to the sheer amount of impervious surface. Cluster 3 cities do have a large amount of low vegetation surrounding the built-up areas of the city; however, they also have a large amount of high vegetation, indicating it is the compact nature of the impervious surface that results in higher averagemaximum LST values. It should be noted that cluster 2 does contain the largest number of cities, which may impact the averages but given the higher averages of cluster 3 and the similarities between the city characterizations, we can assume the data to be consistent.

Given Germany’s long history, the similar characteristics associated with the urban form found in clusters 2 and 3 as well as the resulting higher average maximum LST values is likely a result of historical development, as many Bavarian cities date back to medieval times, with dense centers made of impervious surfaces such as stone with little vegetation. Many of these cities have not expanded significantly when compared with large cities like Berlin. Based on the findings, the city history and evolution has likely impacted the urban form, thereby influencing the LST and therefore must be taken into consideration when compared with newer cities such as those found in Asia or the Americas. It must also be taken into consideration that the background climates and terrain may also impact rural temperatures and consequently the assessment of LST variation [42]. For example, proximity to mountain ranges or other factors associated with varying climate zones may significantly impact the variation in LST, and therefore, requires more detailed future investigation.

### 4.3. Urban interventions

In general, the composition of heat sources and sinks is an essential factor influencing the thermal environment, as the SUHI effect is not equal across cities and it is difficult to remove or significantly change the existing urban form; therefore, the spatial structure of a city requires in-depth analysis for future mitigation planning to better focus on specific areas of a city. Based on the analysis, the findings regarding the impact of urban form on the SUHI effect within German cities are generally consistent with previous studies, particularly the importance of patch shape complexity and dispersal, or fragmentation, in enhancing the cooling effect [20–23]. Therefore, for cities with concentrated areas contributing as heat sources, architectural interventions might become a nessecity. Based on the findings, increased patch shape complexity could potentially alleviate the SUHI effect by increasing air flow between HD urban and surrounding environments. Additionally, cities with high levels of fragmentation and vegetation between urban patches were found to have lower maximum LST values, which confirms the results from existing studies that argue the importance of landscape configuration and particularly the use of separating vegetation [1,25]. Therefore, the implementation of green spaces combined with irregular urban shapes in areas identified as heat sources may assist in the allevation of the SUHI effect.

### 4.4. Limitations of this work

This manuscript is limited to mainly descriptive statistics with the exception of K-means clustering. Hence, the statistical analyses should be completed by more complex models such as neural networks [43], and thorough uncertainty and sensitivity analyses would need to be executed. Additionally, the landscape metrics were chosen based on the findings of previous studies [31,33–35] and availability of the necessary data; therefore, model calibration, apart from the exclusion of certain correlated factors, was not carried out. As such, model comparisons are beyond the scope of this manuscript and are recommended as part of future research.

The statistical approaches presented in the research rely on the initial accuracy of the remote sensing data used. Landsat LST data was chosen due to its worldwide availability and level of detail due to scale, as the analysis focused on cities, rather than entire regions; however, it is important to note that this may not provide the most accurate assessment of the SUHI phenomenon as Landsat data is acquired during morning hours rather than during peak daytime temperatures, limiting the findings. In addition, there are no regular nighttime acquisitions to assess nocturnal heat effects. There are always inaccuracies within data collected from satellite remote sensing as neighboring orbits have time lags of several days, leading to data inconsistencies. These issues are amplified by data gaps due to cloud cover or other atmospheric conditions. Additionally, the 16-day revisit time results in inconsistencies, though the research attempts to mitigate this issue through the collection of data over a 10-year span to avoid as many errors and outliers as possible. Various quantiles were assessed (2.5%, 1%, 0.5%, 0.25%) and it was determined that only 0.5% (top 0.25 and bottom 0.25%) of the data qualified as an outlier.

It is important to note that the pattern analysis is carried out on an urban – non-urban land cover dataset with 10 meter resolution. As such, there are limitations in terms of accuracy as the data cannot represent the urban structure in full detail. Future research utilizing a more detailed dataset may provide additional information on a localized scale for detailed analysis of an individual city. To improve the quality of the satellite data particularly regarding noise and continuous updating of the data basis, recent studies [44,45] have recommended ‘weighting’ or filtering the data to efficiently normalize a dynamic database and increase its accuracy. One potential solution may be the implementation of machine learning methods to produce high-resolution maps and increase data accuracy [46,47]. Additional limitations in data quality must be taken into consideration with regard to the extraction and compilation of data into a database for comparison. The LST data was collected and processed within the terabyte portal and then averaged and extracted into a grid of 100x100m, and as a result, some of the detail is lost in the extraction process. The combination of this data with the additional land cover data and urbanization data allows for analysis of the urban areas for the purposes of the research; however, the extraction process and inconsistency of pixel size among the various datasets reduces the model robustness. To prevent such loss in the future, it may be prudent to implement a fusion method to obtain high-resolution imagery to increase data accuracy, as has been proposed in a recent study by Mishra et al. [48].

The research focuses on identifying potential areas of urban heat stress, rather than thermal comfort. For this research, thermal comfort is defined as the subjective sensation in response to the thermal environment due to deviation from the normal thermal comfort range, while urban heat stress refers to locales of higher than average temperature that negatively impact thermal comfort [2]. Few studies evaluate the potential of LST as a proxy for thermal comfort [2]; however, mapping heat stress has been used in previous research as an established method of identifying vulnerability and risk assessment [49]. Future qualitative analysis regarding general thermal comfort may also provide useful information for urban planners. Additionally, the use of supplementary socio-economic and health data, such as vulnerable population data or hospital admissions, could provide insights into the increasing risk of high temperatures and provide policymakers and planners with additional information to better protect the health of urban residents.

## 5. Conclusion

The results of this research assist in better understanding the relationship between LST and varying urban environmental factors within German cities. In addition, the research highlights the potential of novel data, such as the urbanization level data, as well as the combination of existing methodologies never previously used together for the assessment of urban heat, such as the incorporation of landscape metrics with the traditional statistical analysis. In past studies, a cross-city analysis using varying population ranges was difficult, as existing methods have typically been unable to overcome articifcal boundaries of administrative units for a structurally consistent examination of the intra-urban SUHI phenomenon. The use of the urbanization level data in combination with statistical analysis to measure the variation in LST overcomes this obstacle to compare and measure the extent of the SUHI effect within German cities of various sizes. The use of this methodology in combination with the calculation of landscape metrics to cluster cities based on specific characteristics of urban form enable the study to determine how both city size and urban form may influence the SUHI effect, while also providing insight into potential mitigation strategies. The findings of this work highlight the importance of understanding the relationship between urban form and LST, as well as the capabilities of analysis using remote sensing for future research of urban environments. The main conclusions are as follows:

(i) In German cities, the SUHI effect was detected regardless of size; however, increase in city size does not positively correlate with increase in the SUHI effect; and
(ii) Specific characteristics of urban form (e.g., density and shape) exacerbate the SUHI effect to a greater extent than size in German cities.

While some potential mitigation strategies have been suggested, it should be emphasized that in aiming to alleviate the SUHI effect in German cities, it is important to consider how to best minimize trade offs. Additional sustainability concerns must be taken into consideration, such as reduced GHG emissions; therefore, a case-by-case analysis is recommended over the adoption of an comprehensive measure to alleviate the SUHI effect. By deriving additional knowledge regarding the influencing spatial factors of LST variation and the SUHI effect, we can enable planners and policymakers to take appropriate mitigation measures to reduce potential health risks for urban residents. This research has helped to fill in some of the gaps in existing research and provides a background for future studies to continue adding to the existing knowledge for a more sustainable future.

## Supporting information

S1 Table List of all Cities with Population, Area, and Urbanization Level. (DOCX)

S2 Table City Cluster and Average Mean and Max LST. (DOCX)
