# Supplementary material for: The influence of city size versus urban form on land surface temperature variation and the surface urban heat island effect: A cross-city analysis of German cities
Source: PLoS One. 2026 Feb 10;21(2):e0340060. doi: 10.1371/journal.pone.0340060 (PMC12890098; doi:10.1371/journal.pone.0340060)
Supplement: S1 Table — (DOCX) [file pone.0340060.s001.docx]

**S1 Table. List of all Cities with Population, Area, and Urbanization Level.**

| City | Pop Total | Pop Class | Total Area | % HD | % MHD | % MD | % MLD | % LD |
| --- | --- | --- | --- | --- | --- | --- | --- | --- |
| Berlin | 3,292,365 | GS 1 | 885.32 | 14.49 | 9.80 | 21.12 | 19.86 | 34.74 |
| Hamburg | 1,706,696 | GS 1 | 768.16 | 7.31 | 6.25 | 24.58 | 21.86 | 40.00 |
| Munich | 1,348,335 | GS 1 | 310.27 | 14.91 | 11.47 | 30.63 | 14.30 | 28.69 |
| Cologne | 1,005,775 | GS 1 | 402.93 | 8.04 | 8.05 | 22.49 | 16.73 | 44.69 |
| Frankfurt | 667,925 | GS 1 | 62.47 | 11.70 | 10.95 | 21.19 | 15.03 | 41.13 |
| Düsseldorf | 586,291 | GS 1 | 231.89 | 11.01 | 9.12 | 21.07 | 16.21 | 42.59 |
| Stuttgart | 585,890 | GS 1 | 206.64 | 8.59 | 9.97 | 24.56 | 15.19 | 41.69 |
| Dortmund | 571,143 | GS 1 | 216.31 | 4.32 | 4.45 | 25.43 | 23.49 | 42.31 |
| Essen | 566,201 | GS 1 | 206.82 | 5.91 | 7.45 | 32.00 | 20.88 | 33.75 |
| Bremen | 542,707 | GS 1 | 318.86 | 6.28 | 7.38 | 24.20 | 17.31 | 44.83 |
| Dresden | 512,354 | GS 1 | 278.81 | 2.72 | 4.19 | 11.19 | 18.06 | 63.84 |
| Hannover | 506,416 | GS 1 | 204.02 | 9.33 | 8.75 | 27.72 | 17.40 | 36.81 |
| Leipzig | 502,979 | GS 1 | 150.18 | 14.03 | 10.90 | 22.35 | 23.02 | 29.70 |
| Duisburg | 488,468 | GS 2 | 578.85 | 5.93 | 7.67 | 27.10 | 20.30 | 39.00 |
| Nüremberg | 486,314 | GS 2 | 184.22 | 10.60 | 9.25 | 21.31 | 16.51 | 42.33 |
| Augsburg | 267,767 | GS 2 | 146.94 | 6.05 | 8.59 | 17.51 | 12.04 | 55.81 |
| Regensburg | 135,403 | GS 2 | 80.77 | 7.50 | 11.46 | 22.59 | 17.96 | 40.49 |
| Ingolstadt | 124,927 | GS 2 | 132.79 | 2.92 | 3.44 | 15.11 | 15.42 | 63.12 |
| Würzburg | 124,297 | GS 2 | 67.23 | 5.35 | 6.16 | 19.72 | 17.79 | 50.98 |
| Furth | 115,613 | GS 2 | 248.89 | 5.84 | 6.99 | 23.51 | 19.68 | 43.99 |
| Erlangen | 103,719 | GS 2 | 77.91 | 3.46 | 5.19 | 18.38 | 15.36 | 57.62 |
| Bayreuth | 70,808 | MS 1 | 65.94 | 2.98 | 4.90 | 16.15 | 16.49 | 59.49 |
| Bamberg | 70,635 | MS 1 | 54.91 | 4.60 | 7.35 | 18.54 | 14.08 | 55.43 |
| Aschaffenburg | 67,359 | MS 1 | 63.23 | 3.23 | 4.81 | 15.83 | 13.19 | 62.94 |
| Kempten | 64,078 | MS 1 | 65.26 | 2.04 | 3.17 | 15.10 | 18.59 | 61.10 |
| Landshut | 63,544 | MS 1 | 65.01 | 2.72 | 3.88 | 16.97 | 13.76 | 62.67 |
| Rosenheim | 59,329 | MS 1 | 37.48 | 3.07 | 4.53 | 21.46 | 16.68 | 54.26 |
| Schweinfurt | 52,143 | MS 1 | 36.52 | 7.66 | 8.82 | 22.48 | 13.64 | 47.40 |
| Passau | 48,649 | MS 2 | 67.77 | 1.58 | 1.69 | 13.09 | 25.34 | 58.29 |
| Hof | 44,759 | MS 2 | 57.79 | 3.26 | 4.06 | 16.30 | 13.67 | 62.71 |
| Straubing | 44,488 | MS 2 | 68.35 | 1.75 | 3.51 | 12.08 | 13.46 | 69.20 |
| Amberg | 41,911 | MS 2 | 50.32 | 1.87 | 3.75 | 13.36 | 17.74 | 63.28 |
| Weiden | 41,746 | MS 2 | 87.61 | 1.26 | 2.68 | 10.69 | 14.60 | 70.77 |
| Kaufbeuren | 41,550 | MS 2 | 41.04 | 1.82 | 4.15 | 18.56 | 13.65 | 61.82 |
| Memmingen | 41,241 | MS 2 | 80.62 | 1.32 | 2.57 | 9.62 | 13.53 | 72.96 |
| Coburg | 41,023 | MS 2 | 45.12 | 2.28 | 3.72 | 16.12 | 20.74 | 57.15 |
| Ansbach | 39,491 | MS 2 | 99.99 | 0.75 | 1.68 | 7.09 | 12.21 | 78.28 |
| Schwabach | 38,469 | MS 2 | 40.25 | 1.20 | 2.27 | 15.56 | 20.37 | 60.60 |

HD: High Density, MHD: Medium-High Density, MD: Medium Density, MLD: Medium-Low Density, LD: Low Density

Pop Total based on 2011 census data

Total Area measured in km^2^ based on city administrative boundary using QGIS software for continuity in data results
