# Supplementary material for: The influence of city size versus urban form on land surface temperature variation and the surface urban heat island effect: A cross-city analysis of German cities
Source: PLoS One. 2026 Feb 10;21(2):e0340060. doi: 10.1371/journal.pone.0340060 (PMC12890098; doi:10.1371/journal.pone.0340060)
Supplement: S2 Table — (DOCX) [file pone.0340060.s002.docx]

**S2 Table. City Cluster and Average Mean and Max LST.**

| City | Cluster | Mean LST  Mean (SD) | Max LST  Mean (SD) |
| --- | --- | --- | --- |
| Amberg | 2 | 20.85 (2.785) | 43.67 (4.298) |
| Ansbach | 1 | 20.33 (2.079) | 41.98 (3.892) |
| Aschaffenburg | 2 | 20.28 (2.782) | 40.51 (3.918) |
| Augsburg | 3 | 20.66 (2.758) | 41.67 (4.537) |
| Bamberg | 2 | 21.08 (3.139) | 44.64 (4.609) |
| Bayreuth | 2 | 21.04 (2.619) | 43.49 (3.775) |
| Berlin | 5 | 21.48 (3.022) | 41.49 (5.778) |
| Bremen | 4 | 19.07 (2.478) | 38.35 (4.077) |
| Coburg | 1 | 20.75 (2.592) | 41.95 (3.786) |
| Cologne | 3 | 19.93 (2.496) | 40.86 (4.293) |
| Dortmund | 4 | 19.07 (2.152) | 39.44 (4.14) |
| Dresden | 4 | 20.39 (2.44) | 41.89 (4.743) |
| Duisburg | 4 | 19.96 (2.753) | 39.7 (5.058) |
| Dusseldorf | 3 | 19.72 (2.456) | 39.85 (4.6) |
| Erlangen | 2 | 18.83 (6.282) | 39.38 (13.238) |
| Essen | 4 | 19.17 (2.149) | 39.02 (3.91) |
| Frankfurt | 3 | 21.88 (2.558) | 43.25 (4.08) |
| Furth | 3 | 20.08 (7.138) | 41.21 (13.135) |
| Hamburg | 5 | 19.61 (2.252) | 38.79 (4.15) |
| Hannover | 4 | 21.41 (2.409) | 41.8 (4.251) |
| Hof | 2 | 20.07 (2.324) | 42.98 (3.551) |
| Ingolstadt | 2 | 23.34 (2.815) | 41.34 (5.036) |
| Kaufbeuren | 2 | 18.17 (2.189) | 39.72 (3.594) |
| Kempten | 1 | 17.61 (2.357) | 39.2 (3.987) |
| Landshut | 1 | 22.05 (2.263) | 41.19 (3.833) |
| Leipzig | 4 | 21.69 (2.338) | 42.02 (4.273) |
| Memmingen | 1 | 17.06 (2.154) | 38.84 (4.243) |
| Munich | 3 | 21.28 (2.221) | 40.25 (4.003) |
| Nuremberg | 3 | 21.25 (2.84) | 44.03 (4.222) |
| Passau | 1 | 21.52 (2.385) | 38.79 (3.896) |
| Regensburg | 3 | 24.03 (3.726) | 42.98 (6.451) |
| Rosenheim | 2 | 19.26 (2.238) | 38.76 (4.439) |
| Schwabach | 2 | 20.22 (2.164) | 43.17 (3.606) |
| Schweinfurt | 2 | 23.04 (3.121) | 44.22 (4.925) |
| Straubing | 2 | 23.84 (2.117) | 44.22 (4.241) |
| Stuttgart | 3 | 19.85 (4.086) | 42.12 (7.781) |
| Weiden | 2 | 21.22 (2.87) | 42.63 (4.773) |
| Wurzburg | 2 | 21.75 (3.688) | 43.15 (7.592) |
